# Supplementary material for: Patterns of Intron Gain and Loss in Fungi
Source: PLoS Biol. 2004 Nov 30;2(12):e422. doi: 10.1371/journal.pbio.0020422 (PMC532390; doi:10.1371/journal.pbio.0020422)
Supplement: Table S1 — Also available at http://genes.mit.edu/NielsenEtAl/. (4.3 MB ZIP). [file pbio.0020422.st001.zip › NielsenEtAl/html/735.html]

AN2251.1.NCU09677.1.MG05525.1.FG06575.1


```
 CLUSTAL W (1.82) Multiple Sequence Alignments - Introns Inserted


Sequence 1: NCU09677.1	543 aa
Sequence 2: MG05525.1	512 aa
Sequence 3: FG06575.1	506 aa
Sequence 4: AN2251.1	499 aa
Alignment Length: 575 aa
Number Identitical Residues: 159 aa
Alignment Score (without introns) 8739


MG05525.1 	-----------MAEDGSAAPPAA---------VGAGHAGDAG---------TNSAHDGRG
NCU09677.1	MATIQPPRSSLPAGAEPPAPPSARLDSSDSHTITSTMAGDDGQIDPAQRLPDQQHFNGST
FG06575.1 	------MGDNHSAASGHTATTAVN-----RPTIDTTAATQTQEKLDPTRLTSMDSSTAKR
AN2251.1  	----------MDGGTAHSAPPAD-----------AGHAAAHTDGPAPVQAETRHANNAQG
          	            .    .*..:            :  *    .   .          .  

MG05525.1 	RR---------SGEHLERIATPLSPGATTPPNPFSR-QHTSLDMDDYF~IGPRDTQKHSK
NCU09677.1	EKPRLPRLSIETLPELDAIPTLMSPRHNT-MNPFRR-VHNPLEIDDYF0TGPRDTQKHSK
FG06575.1 	MHS--------LEIPLDNVRSPPSPAVGVNPALFR--KQTSLDLDDYF~TGPRDIQKHSK
AN2251.1  	QHSGPPSEKSSSPGRMTPRPTFLENLADSRDSQFMLNRRDSDDLDRYF0HGPRDLDKHSK
          	 :.  .  . .    :    :  .         *  . : . ::* **  **** :****

MG05525.1 	WPLFMQMHGSILPKLIMPLVFMGLWAGLITSMHM-----------------TDYFKNRGV
NCU09677.1	WPLFMQMHGSILPKMMLPLLGVGAWTTMLTVINFKVTNCALQLSPLPILSRSQAEANIPS
FG06575.1 	WPLVMQMHGSIMPKLIIPLIAIGAWSTAITVIHD-----------------------HYP
AN2251.1  	WPIMMRMHGSIMPKMILPITFVAGWSTCITLISR-----------------------FVY
          	**:.*:*****:**:::*:  :. *:  :* :                            

MG05525.1 	RV1SVNSVLLTVLGFVVGLGLSFRSSTAYER~YAEGRRYWASLILASQSLGRVFWIHTRE
NCU09677.1	PV~GIHNILLTVLGFVVGMGLSFRSSTAYER~YSEGRRYWASLLLACQTLGRVYWVHAKE
FG06575.1 	KI~QVNSVLLTILGFVVGLSLSFRSSTAYER~YAEGRRYWGTLTMASQTLGRVIWIHGKD
AN2251.1  	RL1SVDNILLTVLGFVVGLSLSFRSSTAYER2WADGRKYWSLLVQTSRNLSRTIWINTLE
          	 :  :..:***:******:.*********** :::**:**. *  :.:.*.*. *::  :

MG05525.1 	RD-ETREKDILNKL~SALNLLTAFAVALKHKLRFEPYTNYDDLSQMVGHLDTLAGEAT--
NCU09677.1	REGELGKKDVLAKL2TVLNLLVAFSVALKHRLRFEPYTCYDDISHLVAHLDTFAQRATEE
FG06575.1 	VPDQDPRETILKKI~GAMNLIVAFAVSLKHALRFEPYSAYPDMEHLIGHLNTFAKDAT--
AN2251.1  	REGELGKEDLLRKL2TAINLILAFAVALKHKLRFEPDVGYEDLAGLVGYLDTFAKEAH--
          	  .:  .: :* *:  .:**: **:*:*** *****   * *:  ::.:*:*:*  *   

MG05525.1 	KTDGGATAVKNPNIFKRFGSMLGVPFCESNPRKQLKKAKRPLGNLPLEILNYLAAYTDEI
NCU09677.1	EPDKTLKSLQPPGFFKAVGEYIGVSFAASNPRKVIKKASRPLGNLPLEILCYLATYTDEL
FG06575.1 	LADPDAGSMKKKNIFKSVGEYLGVSFAQSNPRKALKKTDKPLGNLPLEILSHLAVTIDRM
AN2251.1  	--DRQVIQPRKRTIWKVTGEYLGVSFAESNPRKLIKRSKKPLGHLPLEILNHLSAYIDSV
          	  *      :   ::*  *. :**.*. ***** :*::.:***:****** :*:.  * :

MG05525.1 	VANGQLPVPMQQTLA1Y~NNMALLNDVMVGTERVLSTP0LPIAYSIAIAQITWLYVFLLP
NCU09677.1	IANGQLPVPMQQTLA~Y~NNMAILNDVLVGTDRVLSTP~LPIAYAIAIAQITWVYILLLP
FG06575.1 	VAQGQLDIPMQQTLA~Y~NNLGLMNDCMTGCDRVLNTP~LPIAYTIAISQITFIYIFVLP
AN2251.1  	IRNETLNISLHQSQA~I1ACLAQLNEVVTGTERVLDTP~LPVAYSIAIAQIAWIYVLVLP
          	: :  * :.::*: *     :. :*: :.* :***.** **:**:***:**:::*:::**

MG05525.1 	FQLCATLEWVAIPATVAASYIILGLLYIGREI2EQPFGLDPNDLPLESYCAQIAAELDVL
NCU09677.1	FQLLPTLNWITIPATIAAAYIILGLLMIGREI~ENPFGRDVNDLPLESYCAQVAEDMDII
FG06575.1 	FQLVQTLEWITIPASIVAAYIIFGLLFIGQEI~ENPFGQDVNDLPLEIYCDQIAADLDVI
AN2251.1  	FQLYDSLGWVTIPGSIVAAYIILGLATIGSEI~ENPFGHDVNDLPLDTYCRQIALELDIV
          	***  :* *::**.::.*:***:**  ** ** *:*** * *****: ** *:* ::*::

MG05525.1 	ASRPK-PRISEWTEHLENKVLFPYSNAGWHAWLHHGETKIRQALRAKTELEFGQRSRMMG
NCU09677.1	ACQKKRTTNADWIENIDNKPLWPLSNSGWQVWMNRGEDMIREAVHQKLESNFETR-----
FG06575.1 	ASHDK-REPDTFLLSHNSMPLYPVSTASPNAWMKRSDEKLRQVIKDKPNTMFEWR-----
AN2251.1  	TAVPP-PSVDEFTSREDNYVLYPLSTSGQPEWKERSVEDIRAALRAKVVANTSPTAS---
          	:.         :    :.  *:* *.:.   * .:.   :* .:: *        :    

MG05525.1 	QEHGKNEGGSGHAHGHGRSVSKP-QAAEKISDV~ENGLGSRQ--
NCU09677.1	-----------------KELDKL-QSEMSLMDK0RVV-------
FG06575.1 	-----------------KEIARKKQSGAKFGTN~DMMTPGDHNV
AN2251.1  	-----------------KTSTVLEEVNTKLNKQ~SSV-------
          	                 :     .:   .:
```
